# Development of a distributed nonlinear Muskingum model by considering snowmelt effects for flood routing in the Red River

**DOI:** 10.1038/s41598-023-48895-8

**Published:** 2023-12-04

**Authors:** Vida Atashi, Reza Barati, Yeo Howe Lim

**Affiliations:** 1 Department of Civil Engineering, University of North Dakota, Upson II Rm. 260K, 243 Centennial Dr. Stop 8115, Grand Forks, ND 58202-8115 USA; 2 Department of Civil Engineering, Tarbiat Modares University, Tehran, Iran; 3 Department of Civil Engineering, University of North Dakota, Upson II Rm. 260D, 243 Centennial Dr. Stop 8115, Grand Forks, ND 58202-8115 USA

**Keywords:** Hydrology, Natural hazards

## Abstract

This research paper presents the development of a nonlinear Muskingum model which achieves precise flood routing through river reaches while considering lateral inflow conditions. Fourteen pairs of flood hydrograph found at two specific United States Geological Survey (USGS) stations located along the Red River of the North, namely Grand Forks and Drayton, are used for the calibrations and validations of the Muskingum model. To enhance the accuracy of the procedure, a reach is divided into multiple sub-reaches, and the Muskingum model calculations are performed individually for each interval using the distributed Muskingum method. Notably, the model development process incorporates the use of the Salp Swarm algorithm. The obtained results demonstrate the effectiveness of the developed nonlinear Muskingum model in accurately routing floods through the very gentle river with a bed slope of (0.0002–0.0003). The events were categorized into three groups based on their dominant drivers: Group A (Snowmelt-driven floods), Group B (Rain-on-snow-induced floods), and Group C (Mixed floods influenced by both snowmelt and rainfall). For the sub-reaches in Group A, single sub-reach (NR = 1), the Performance Evaluation Criteria (PEC) yielded the highest value for SSE, amounting to 404.9 × 10^6^. In Group B, when NR = 2, PEC results the highest value were SSE = 730.2 × 10^6^. The number of sub-reaches in a model has a significant influence on parameter estimates and model performance, as demonstrated by the analysis of hydrologic parameters and performance evaluation criteria. Optimal performance varied across case studies, emphasizing the importance of selecting the appropriate number of sub-reaches for peak discharge predictions.

## Introduction

Flood routing is a process of simulating the movement of water in a river or stream system during a flood event using mathematical models. The goal of flood routing is to predict the behavior of the water as it moves through the system, including the peak flow, the timing of the peak flow, and the overall duration of the flood. There are two basic approaches to routing flood waves in natural channels: hydrologic (lumped) and hydraulic routing. Hydrologic (lumped) routing is a simplified approach that treats the entire river or stream system as a single unit. This approach relies on the storage continuity equation, which states that the change in storage in a system is equal to the difference between the inflow and outflow. Hydraulic routing is a more complex approach that considers the physical characteristics of the river or stream system, such as the channel geometry, the roughness of the bed, and the presence of any structures. Hydrologic routing is typically used for flood forecasting and planning, while hydraulic routing is more commonly used for the design and operation of flood control structures^1, 2^. The Muskingum model is a widely accepted hydrologic routing model due to its adequate levels of accuracy and the reliable relationships between its parameters and channel properties.

## Muskingum model

The Muskingum method is founded on the fundamental principles of mass and momentum conservation, positing that the discharge at a given point in the river can be derived by subtracting the outflow from the inflow^3^. This model uses a linear reservoir approach to model the river channel characterized by two parameters, namely, the wave travel time (*K*) and the reach weighting factor (*x*).

The travel time (*K*) is the time required for the water to travel through the reach, which is dependent on the channel geometry, roughness, and other hydraulic characteristics. The reach weighting factor (*x*) is the proportion of the discharge that enters the reach from the upstream section, which is also known as the weighting coefficient. These parameters can be determined using various techniques, including trial and error, optimization algorithms, and regression analysis. The Muskingum model can be represented mathematically as follows^4^:

1 $$S=K\left[xI+\left(1-x\right)O\right]$$

where *O* is the discharge at the downstream end of the reach (m^3^/s), and *I* is the discharge at the upstream end of the reach (m^3^/s). *x* is the weighting factor for the reach (ranges between 0 and 0.5 for reservoir storage and between 0 and 0.3 for stream channels^5^), *K* is the travel time for the reach(s), and *S* is the storage volumes of the reach (m^3^). By combining Eq. (1) with the continuity equation an explicit equation can be obtained to calculate the outflow at the next time step:

2 $${O}_{2}={C}_{0}{I}_{2}+{C}_{1}{I}_{1}+{C}_{2}{O}_{1}$$

The subscripts 1 and 2 on *I* and *O* represent the values at time *t*_1_ and *t*_2_ respectively. *C*_0_, *C*_1,_ and *C*_2_ are the coefficients.

The traditional linear Muskingum model seeks a method of parameter estimation to determine the values of K and x. However, the linear Muskingum model leads to considerable inaccuracy in the forecast of flood behavior throughout its propagation along a river because natural channel reaches often have a nonlinear storage-discharge connection. To address this limitation, models such as the Muskingum model have been modified to account for the nonlinearity of flow movement processes. Gill^6^ introduced a nonlinear storage equation using the exponent of the Muskingum storage equation as the third parameter, and later models such as the Nonlinear Muskingum model (NLMM) have been developed to include lateral inflows and better simulate the nonlinear processes of flood movements in rivers. As stated by Perumal et al.^7^ there exists no “truly physically based” flood routing model which does not require any calibration. Although the roughness factor is a property of the natural conditions, it is considered as a model tuning parameter in the routing process of Muskingum–Cunge models^8, 9^.

Furthermore, as pointed out by Koussis, nonlinear routing models like the nonlinear Muskingum model possess an advantage in their ability to accurately replicate the rapid surge of a flood wave, a task that linear models often struggle with^10, 11^. It should be noted that the Muskingum–Cunge is not a “linear model”. However, it is a “time-variant linear model”, which means that it is “locally linear” in time, but the overall behavior is nonlinear. Every flood routing model necessitates specific input parameters and data. In some river segments, all the required inputs are readily available, and the choice of a model can be based on personal expertise and computational capacity. When there are no constraints on these factors, the use of a dynamic wave model is the most suitable option. For the Muskingum–Cunge model, essential input parameters include initial conditions, upstream boundary conditions, Manning's roughness coefficient, length of the routing reach, river cross-sections, and the bed slope^12^, while nonlinear Muskingum model requires the initial condition, upstream boundary condition and the hydrologic parameters. One of the important motivations of the authors is to suggest alternative hydrological flood routing model for using in modeling software of hydrologic processes of watershed systems such as HEC-HMS (hydrologic modeling system) and SWAT (soil and water assessment tool). The proposed and rigorously validated distributed hydrological Muskingum model can serve as a valuable addition to hydrological software, effectively mitigating uncertainties in flood modeling. It's important to note that this study primarily emphasizes the nonlinear Muskingum routing models.

## Nonlinear Muskingum model

Previous research has advocated a nonlinear Muskingum model for accounting nonlinearity, which allows for a better representation of the nonlinear relationship between the inflow at the upstream end and outflow at the downstream end of the river channel, which is presented in Eq. (3)^5, 6, 13–15^:

3 $$S={K\left[x{I}_{t}+\left(1-x\right){O}_{t}\right]}^{m}$$

where *m* takes the nonlinearity without lateral inflow to the models. These models feature an extra parameter *m* (= exponent power), which may be calculated using various parameter estimation approaches. On the other hand, *K* with dimension of ${L}^{3(1-m)}{T}^{m}$ in nonlinear models unlike the linear model does not describe the travel time of the flood wave. In addition, *x* does not have to be the same as in the linear model. Equation (4) shows a modified storage equation that considers lateral inflow^16^:

4 $$\frac{dS}{dt}=\frac{\Delta S}{\Delta t}=\left(1+\beta \right){I}_{t}-{O}_{t}$$

where *β* is the parameter accounting for the lateral inflow. The storage at time *t* + *1* is shown in equation below.

5 $${S}_{t+1}={S}_{t}+\Delta S$$

By substituting Eq. (4) into Eq. (5), with consideration of lateral inflow in a nonlinear relationship between the inflow at the upstream end and outflow at the downstream end, the storage at time *t* + *1* is represented in Eq. (6):

6 $${S}_{t}=K{\left[\left(1+\beta \right)x{I}_{t}+\left(1-x\right){Q}_{t}\right]}^{m}$$

The NLMM with the lateral inflow (*NLMM-L*) has been suggested as an accurate solution method for addressing the nonlinear Muskingum model^17–26^. The Muskingum model can be solved using various numerical methods, with the fourth-order Runge–Kutta method standing out as one of the most accurate approaches. Here, the fourth order Runge–Kutta method has been offered as an accurate and acceptable solution method among the different explicit solution methods for addressing the nonlinear Muskingum model since it is simpler than the Runge–Kutta–Fehlberg method^27, 28^.

It's important to note that Cunge^12^ established a vital connection between the flood routing parameters within the Muskingum approach and the channel properties, as well as flow characteristics. This connection was achieved by utilizing an approximation error derived from a Taylor series expansion of grid specifications and employing a diffusion analogy. Consequently, Cunge introduced a model known as the Muskingum–Cunge model, which has served as a cornerstone for further research and refinement^7, 29–35^.

This particular class of flood routing models necessitates a set of crucial input parameters, including the initial condition, upstream boundary condition, Manning's roughness coefficient, length of the routing reach, cross-sections along the river reach, and the bed slope. Within these inputs, the Manning's roughness coefficient stands out as a notable source of uncertainty for Muskingum–Cunge models. This coefficient is closely linked to surface roughness, vegetation, channel irregularities, channel alignment, silting, scouring, obstruction, channel size, and shape, as well as the magnitudes of stages and discharges^36^. Most of these factors exhibit variations from one flood event to another within a given river reach^9^. As a result, the roughness variations within a river reach are inherently three-dimensional, making them challenging to model. Therefore, there's a need to strategically select a single parameter as the roughness coefficient through a calibration process to align the flood routing results, particularly when only a single set of inflow and corresponding outflow hydrographs are available for the considered river reach^7, 8^.

In contrast, the traditional linear Muskingum model primarily relies on the initial condition, upstream boundary condition, and various hydrologic parameters^10, 11^. One of the principal motivations of the authors is to propose an alternative hydrological flood routing model suitable for integration into modeling software for hydrologic processes within watershed systems like HEC-HMS (hydrologic modeling system) and SWAT (soil and water assessment tool). Consequently, the primary focus of this study revolves around enhancing nonlinear Muskingum routing models.

To further improve its accuracy and convergence, optimization algorithms like the Salp Swarm algorithm have emerged as effective tools. Salp Swarm algorithm is a population-based optimization algorithm inspired by the swarming behavior of salps. The algorithm starts with a population of salps, each of which has a random position in the search space. In each iteration, the salps move towards the leader salp, which is the salp with the best fitness^37^. The salps that have the best fitness values are more likely to be selected for reproduction, and their offspring are added to the population. This process continues until the algorithm converges on an optimal solution^38, 39^. SSA has been shown to outperform other optimization algorithms in terms of both accuracy and convergence speed^37^ and has the potential to be used to solve a wide variety of problems^40, 41^.

Through the integration of the Salp Swarm algorithm and *NLMM-L* Muskingum method, the research seeks to address the challenges associated with snowmelt-induced flooding and provide more precise flood routing solutions for the study area. The objective of this research is to develop a nonlinear Muskingum model for the Red River between two USGS stream gauging stations, Grand Forks, and Drayton, in the US, using flood hydrographs caused by snowmelt in spring. This area was selected due to the recurrent flooding observed in the central part of the Red River and its adjacent floodplain regions, stretching between Grand Forks, ND, and Emerson, ND. These flood events are notably characterized by the substantial seasonal water area that consistently forms during wet spring periods^42^. The flat terrain and downstream ice jams in the Red River and Lake Winnipeg contribute to the frequent flooding during spring seasons in wet years, including 1997, 2009, 2011, and 2013. The repetitive nature of flood events in this section of the Red River underscores the importance of comprehending and effectively managing flood risks in the region.

The primary objective of this study is to develop a Muskingum model that can accurately estimate river discharge, considering lateral inflow conditions. The research methodology involves estimating the parameters (*K*, *x*, *m*, and *β*) of the nonlinear Muskingum models using a distributed flood routing model, utilizing the Salp Swarm algorithm. This approach divides the river reach into multiple intervals, allowing individual Muskingum model calculations for each interval, thereby improving the overall accuracy of the estimation process. The findings of this research are expected to contribute to the enhancement of flood forecasting and warning systems in the Red River basin, enabling better preparedness and response to flood events.

## Study area

**Figure 1:**
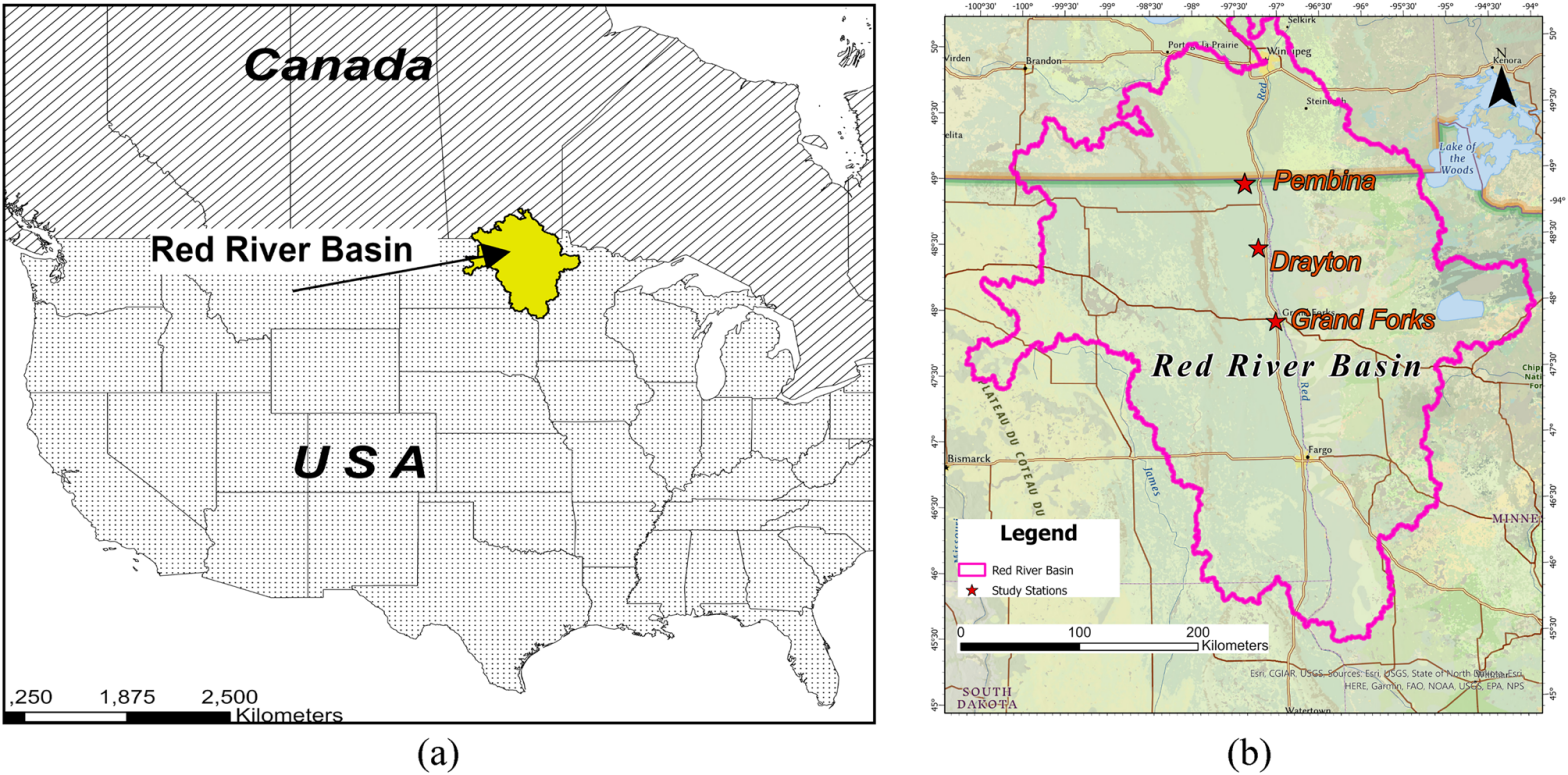
(**a**) Red River basin (**b**) USGS stations on Red River in Drayton and Grand Forks^51^. The map was created using ArcGIS Pro 2.8.0 (https://www.esri.com/en-us/arcgis/products/arcgis-pro/overview).

The Red River Basin, spanning both the United States and Canada, encompasses an area of 117,185 km^2^, with most of its expanse situated in North Dakota, South Dakota, and Minnesota^43^. Figure 1a shows the location of the basin. Characterized by a semi-arid climate, the region experiences cold winters and hot, dry summers, with the primary streamflow occurring during spring and early summer due to snowmelt or heavy rainfall^44^. The Red River itself is a prominent watercourse within the basin, flowing northward along the border between Minnesota and North Dakota, as well as the Canadian provinces of North Dakota and Manitoba. Notably, the river is prone to frequent flooding, owing to its gentle flow and flat topography, and its broad and shallow floodplain exacerbates the vulnerability to heavy rain or spring snowmelt, resulting in historically devastating floods^45–49^.

A recent study by Atashi et al.^50^ investigated various forecasting methods for water levels in flood warning systems. The study found that the Long Short-Term Memory (*LSTM*) method demonstrated superior accuracy and precision compared to classical statistical and machine learning approaches, making it a reliable choice for flood prediction, particularly for downstream stations lacking discharge information^50^.

## Methodology

### Grouping of dataset

For conducting flood routing analysis, it is essential to have observed flow hydrographs at specific upstream and downstream cross-section pairs.

For this study, we selected the existing USGS streamflow gauging stations at Drayton (Station No. 05092000) and Grand Forks (Station No. 05082500). These stations were chosen because they offer vital streamflow data necessary for hydrograph analysis in the region extending from Grand Forks to the US-Canada border. As mentioned, this area has experienced recurrent flooding, particularly in the central part of the Red River and the nearby floodplain regions, which extend from Grand Forks, ND, to Emerson, ND. The locations of the gauging stations are shown in Fig. 1b.

A total of fourteen flood events occurring between 1990 and 2022 were utilized to calibrate and validate the proposed model, with twelve events designated for calibration and two events for validation. These validation events specifically corresponded to the flood events in 2020 and 2022.

To form the groups, specific criteria were considered based on the distinct routing characteristics observed in different flood types. For instance, the 1997 flood primarily resulted from snowmelt with minimal rain-on-snow impact, while the 2022 flood predominantly comprised rain-on-snow conditions. The selection of these criteria was crucial to ensure accurate calibration results across all events. The summarized data for the fourteen flood occurrences between 1990 and 2022 is presented in Table 1, with the events categorized into Group A, Group B, and Group C. The criteria used for forming the groups include:

1. Snowmelt dominant events: flood events where the primary driver was snowmelt with minimal rain-on-snow impact were categorized into Group A. These events typically exhibit specific routing characteristics associated with snowmelt-dominated hydrological processes.
2. Rain-on-snow dominant events: flood events predominantly characterized by rain-on-snow conditions were categorized into Group B. These events show distinct routing behavior resulting from the combined effects of rain and snowmelt on the hydrological system.
3. Mixed events: flood events that had a combination of snowmelt and rain-on-snow conditions were categorized into Group C. These events exhibit routing characteristics influenced by both snowmelt and rainfall contributions.

**Table 1:** Characteristics of the spring snowmelt flood events at two Red River stream gauging stations.

Flood groups	Years	No. of floods’ occurrence	Frequency of data
A	1997, 2001, 2005, 2006, 2009, 2010, 2018, and 2020	8	Daily
B	1999, 2004, 2013, and 2022	4	Daily
C	2011 and 2019	2	Daily

By categorizing the flood events into these distinct groups, it was possible to calibrate the model separately for each flood type, considering the specific routing behaviors associated with each category. Group A and Group B exhibit distinct variations in terms of precipitation amounts. Specifically, Group A demonstrates a lower range of monthly precipitation, ranging from 57. 2 to 141.0 mm during the selected years. In contrast, Group B showcases a higher range of monthly precipitation, ranging from 187.3 to 276.4 mm, observed specifically in the years 1999, 2004, 2013, and 2022.

### Distributed nonlinear Muskingum model incorporating lateral inflows

**Figure 2:**
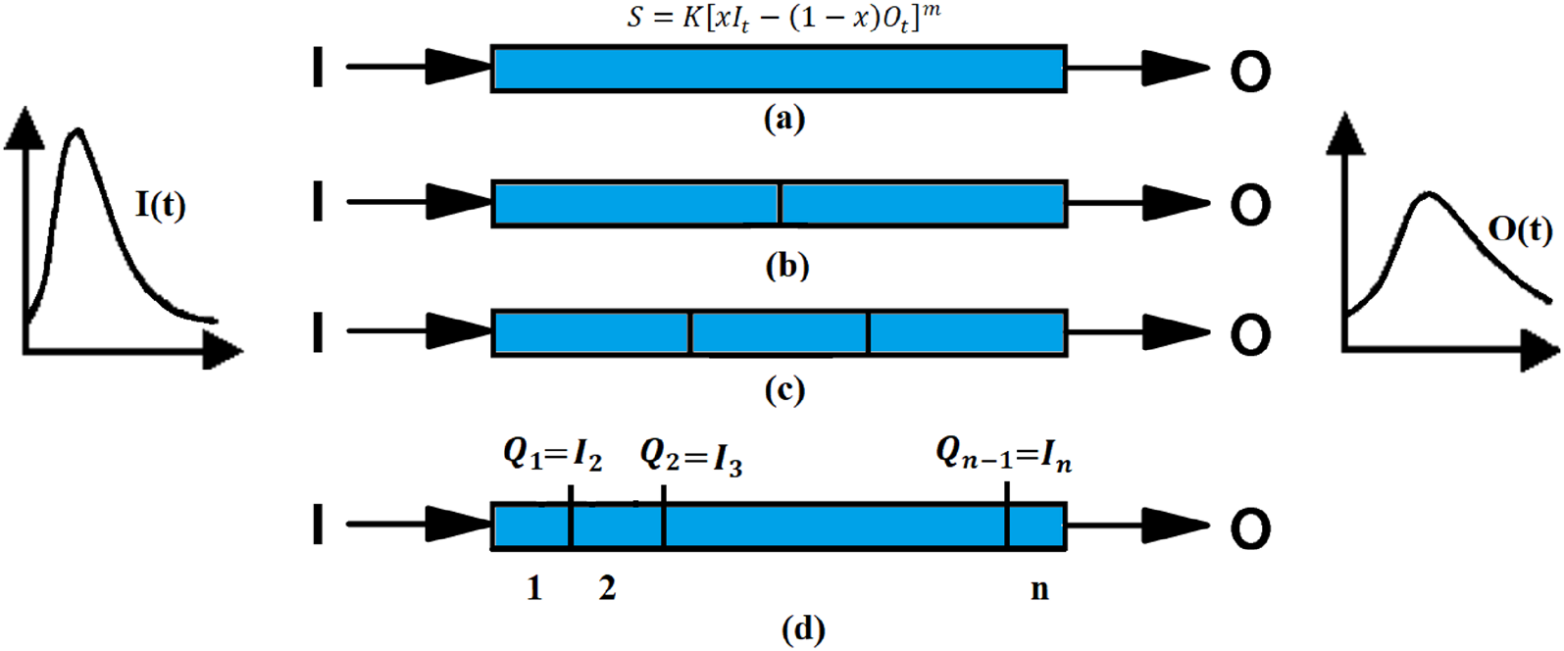
Models for distributed nonlinear Muskingum model: (**a**) single reach with no sub-reaches, (**b**) two sub-reaches within a reach, (**c**) three sub-reaches within a reach, and (**d**) multi-interval sub-reach within a reach.

Nonlinear Muskingum models consist of a series of nonlinear Muskingum reaches, which are further subdivided into equal nonlinear Muskingum sub-reaches. The distributed nonlinear Muskingum model, as depicted in Fig. 2, provides an illustrative representation of this arrangement. Only one set of hydrological model parameters (*K*, *x*, and *m*) needs to be calibrated and used in the nonlinear routing calculations. The flood hydrograph is routed from the main inflow hydrograph at the upstream section to the downstream section of the first sub-reach. The outcome is treated as the inflow for the second sub-reach and is routed subsequently to the downstream section of the second sub-reach^52^. To get the flood hydrograph at the downstream section of the final sub-reach, this process is repeated sequentially. The number of sub-reaches (*NR*) can be determined by trying different options and selecting the one that gives the best results. An objective function value and other performance evaluation criteria can be used to compare the different *NR* options. The continuity and storage equations used in the distributed nonlinear Muskingum model that includes lateral inflows are presented as follows:

7 $$\frac{d{S}_{t}^{j}}{dt}=\left(1+\beta \right){Q}_{t}^{j-1}-{Q}_{t}^{j}$$

8 $${S}_{t}^{j}={K\left[\left(1+\beta \right)x{Q}_{t}^{j-1}+\left(1-x\right){Q}_{t}^{j}\right] }^{m}$$

where the lateral inflows varied linearly along the river reach and could be represented as a ratio of the inflow rate by considering the *β* parameter. *β* allows for the consideration of lateral inflow or outflow from the main channel during flood events. It represents the ratio of the inflow or outflow to the main channel flow within the reach. One of the assumptions used in the modeling process is that *β* is constant in time that means hydrograph shape of the lateral inflow wave is proportional to the upstream hydrograph inflow. *t* is the measure of time between zero and the flood's finish time. The spatial index between 2 and *NR* + *1* is called *j*. The following stages are used in the routing strategy for the distributed nonlinear Muskingum model utilizing the fourth order Runge–Kutta method:

1. Choose one, two, three, or more sub-reaches as *NR* and assume random values for the hydrological model parameters *K*, *x*, and m, as well as $\beta$.
2. Use Eq. (8) to estimate the starting storage. The starting flow rate at each sub-reach's downstream part is the same as the initial flow rate at the sub-reach's upstream section. Calculate the next storage.
3. The next storage is computed by the present value plus the product of the size of the interval, *Δt*, and an estimated slope. The slope will be a weighted average of the following slopes using the Fourth order Runge–Kutta method:
  9 $${{L}_{1}}_{t}^{j}=-\left(\frac{1}{1-X}\right){\left(\frac{{S}_{t}^{j}}{K}\right)}^{1/m}+\left(\frac{1+\beta }{1-X}\right){Q}_{t}^{j-1}$$
  10 $${{L}_{2}}_{t}^{j}=-\left(\frac{1}{1-X}\right){\left(\frac{{S}_{t}^{j}+0.5{{L}_{1}}_{t}^{j}\Delta t}{K}\right)}^{1/m}+\left(\frac{1+\beta }{1-X}\right)\left(\frac{{Q}_{t}^{j-1}+{Q}_{t+1}^{j-1}}{2}\right)$$
  11 $${{L}_{3}}_{t}^{j}=-\left(\frac{1}{1-X}\right){\left(\frac{{S}_{t}^{j}+0.5{{L}_{2}}_{t}^{j}\Delta t}{K}\right)}^{1/m}+\left(\frac{1+\beta }{1-X}\right)\left(\frac{{Q}_{t}^{j-1}+{Q}_{t+1}^{j-1}}{2}\right)$$
  12 $${{L}_{4}}_{t}^{j}=-\left(\frac{1}{1-X}\right){\left(\frac{{S}_{t}^{j}+{{L}_{3}}_{t}^{j}\Delta t}{K}\right)}^{1/m}+\left(\frac{1+\beta }{1-X}\right){Q}_{t+1}^{j-1}$$

By weight averaging these four slopes, one can calculate the next storage by using the following equation:

13 $${S}_{t+1}^{j}={S}_{t}^{j}+\frac{\Delta t}{6}\left({{L}_{1}}_{t}^{j}+2{{L}_{2}}_{t}^{j}+2{{L}_{3}}_{t}^{j}+{{L}_{4}}_{t}^{j}\right)$$

14 $${Q}_{t+1}^{j}=\left(\frac{1}{1-X}\right){\left(\frac{{S}_{t+1}^{j}}{K}\right)}^{1/m}-\left(\frac{X}{1-X}\right)\left(1+\beta \right){Q}_{t+1}^{j-1}$$

4. Calculate the next outflow by using the following equation:
5. Repeat Steps 3 and 4 for the following time intervals.
6. Repeat Steps 2 and 5 for subsequent sub-reaches.

### Salp swarm algorithm (SSA)

The Salp swarm algorithm (*SSA*) is a population-based swarm intelligence algorithm developed in 2017 by Mirjalili et al.^37^. The food source, which is the objective of the swarm, is represented by *F*. The leader of the swarm updates its position using a specific equation below:

15 $${x}_{j}^{1}=\left\{\begin{array}{c}{F}_{j}+{c}_{1}\left(\left(U{b}_{j}-l{b}_{j}\right){c}_{2}+l{b}_{j}\right) {c}_{3}\ge 0\\ {F}_{j} - {c}_{1}\left(\left(U{b}_{j}-l{b}_{j}\right){c}_{2}+l{b}_{j}\right) {c}_{3}<0\end{array}\right.$$

where ${x}_{j}^{1}$ is the position of leader in *j*th dimension, $U{b}_{j}$ are the upper and lower boundary at *j*th dimension, ${F}_{j}$ is the food source position. The coefficient ${c}_{1}$ plays an important role in *SSA* balancing exploration and exploitation. During the process of optimization, exploration refers to searching the search space thoroughly to find better solutions, while exploitation refers to utilizing the information present in the local region to improve the current solution. The parameter ${c}_{1}$ is gradually decreased over iterations and can be calculated using the following formula.

16 $${c}_{1}=2{e}^{{-\left(\frac{4t}{L}\right)}^{2}}$$

where *l* is the current iteration and *L* is the maximum number of iterations. The parameters ${c}_{2}$ and ${c}_{3}$ are random numbers generated within the interval [0,1]. ${c}_{3}$ is responsible for indicating whether the next position of current leader salp should be toward + ∞ or -− ∞. The other members of the salp swarm update their positions based on Newton's law of motion, which is expressed using the following equation:

17 $${x}_{j}^{i}= \frac{1}{2}a{t}^{2}+{v}_{0}^{t}$$

where $i\ge 2$, ${x}_{j}^{i}$ is the position of the *i*th follower in the *j*th dimension, *t* is the time, ${v}_{0}$ is the initial speed, and $a= \frac{{v}_{final}}{{v}_{0}}$ where $v=\left(x-{x}_{0}\right)/t$.

Since the time is considered as iterations and ${v}_{0}=0$, Eq. (15) can be reformulated as the equation below:

18 $${x}_{j}^{i}=\frac{1}{2}({x}_{j}^{i}+{x}_{j}^{i-1})$$

where $i\ge 2$, ${x}_{j}^{i}$ is the position of the *i*th follower in the* j*th dimension.

**Figure 3:**
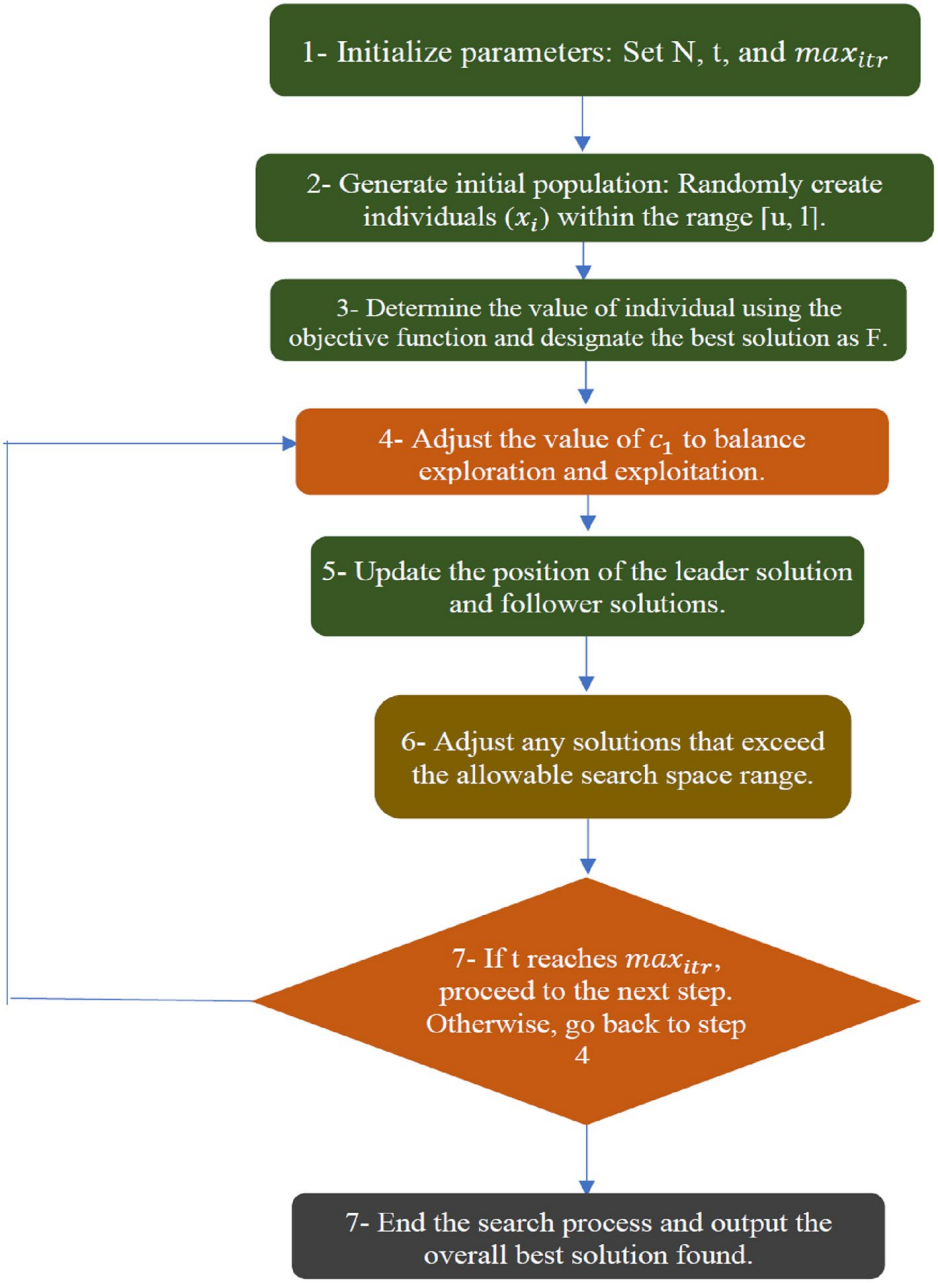
Optimization algorithm flowchart for salp swarm algorithm (SSA).

The main steps of the *SSA* can be summarized as follows (see Fig. 3):

- *Parameter initialization:* the algorithm starts by initializing the parameters such as the population size *N*, number of the iterations *t* and the maximum number of iterations ${max}_{itr}$.
- *Initial population:* we generate the initial population ${x}_{i}$, $i=\left\{1, ..., n\right\}$ randomly in the range [u,l], where *u* and l are the upper and lower boundaries, respectively.
- *Individual evaluations:* every individual (solution) within the population is assessed by determining its value using the objective function, and the best overall solution is designated as *F*.
- *Exploration and exploitation:* to balance between the exploration and the exploitation of the algorithm, the value of the parameter ${c}_{1}$ is updated as shown in Eq. (16).
- *Update the position of the solutions:* the position of the leader solution and the other follower solutions are updated as shown in Eqs. (15) and (18), respectively.
- *Boundary violations:* boundary violations occur when a solution goes beyond the allowable range of the search space while updating, and it is then adjusted to fall within the problem's range.
- *Termination criteria:* the number of iterations t is increased gradually until it reaches to the maximum number of iterations ${max}_{itr}$. Then the algorithm terminates the search process and produces the overall best solution found.

### Statistical performance evaluation criteria

Statistical performance evaluation criteria are metrics used to assess the accuracy and reliability of mathematical models, such as hydrological or hydraulic models. Some common statistical performance evaluation criteria used in the papers referenced^5, 14, 53–59^ are applied to assess the performance of the SSA-based routing results. These criteria are described below.

Sum of squared errors (*SSE*): the *SSE* measures the sum of the squared differences between predicted and observed values. It measures the model’s overall error and indicates how well the model fits the observed data.

19 $$SSE={\sum }_{t=1}^{N}{\left\{{O}_{t}-{\widehat{O}}_{t}\right\}}^{2}$$

where ${Q}_{i}$ and ${\widehat{Q}}_{\upiota }$ respectively are the observed and calculated outflow rates at the *i*th time, and *N* is the number of data.

The sum of absolute differences (*SAD*): the *SAD* measures the sum of the absolute differences between predicted and observed values. It is a measure of the overall deviation of the model from the observed data and is useful for evaluating the model's performance under conditions where large errors may have a significant impact.

20 $$SAD={\sum }_{t=1}^{N}\left|{O}_{t}-{\widehat{O}}_{t}\right|$$

Difference of peak observed (*DPO*): the *DPO* measures the difference between the predicted discharge values from the observed peak discharge values. It is a measure of the model's ability to accurately predict extreme events, such as floods or droughts, that may have a significant impact on the environment or society^53^.

21 $$DPO= \left|{Peak}_{routed}-{Peak}_{Observed}\right|$$

The deviation of peak time of routed and actual outflows (*DPOT*).

22 $$DPOT=\frac{\left|{T}_{\text{pest}}\hspace{0.33em}-{\text{T}}_{\text{pobs}}\right|}{\Delta t}$$

${T}_{pobs}$ and ${T}_{pest}$ denote the observed and estimated times to peak discharge, respectively. All the criteria presented are measurements of the accuracy of a routing model, with the optimum value at 0.

## Results

Table 2 presents estimates of hydrologic parameters and performance evaluation criteria (*PEC*) values for Group A, considering different numbers of sub-reaches. The table encompasses sub-reach configurations ranging from 1 to 4, each characterized by Muskingum parameters (*k*, *x*, *m*, and *β*). The performance evaluation criteria include *SSE*, *SAD*, *DPO*, and *DPOT*.

**Table 2:** Hydrologic parameters estimates and PEC values for different numbers of sub-reaches applied for Group A.

Number of sub-reaches	Model parameters				PEC			
	*x*	*K*	*m*	*β*	*SSE*	*SAD*	*DPO*	*DPOT*
1	**0.24**	**0.54**	**1.38**	**0.19**	404,942,172.92	111,755.01	2,686.62	0
2	0.08	0.35	1.24	0.09	524,984,042.89	136,482.56	608.52	1
3	0.09	0.20	1.26	0.05	679,360,429.21	157,998.12	1,030.29	1
4	0.14	0.12	1.27	0.04	774,514,517.49	169,371.14	1,958.94	1

Significant values are in [bold].

The table clearly demonstrates that the number of sub-reaches employed has a substantial influence on both the estimates of hydrologic parameters and the corresponding *PEC* values. Analysis of the performance criteria indicates that the optimal performance is achieved when the Red River at Drayton station is considered as a single sub-reach. This is primarily due to the single sub-reach model exhibiting the lowest SSE values. Using the current study algorithm for Group A flood, the optimized parameters were determined to be *K* = 0.54, *x* = 0.24, *m* = 1.478, and *β* = 0.19 for *NR* = 1.

The hydrologic parameters estimates and performance evaluation criteria (*PEC*) values for Group B were investigated using various numbers of sub-reaches, ranging from 1 to 4. Each sub-reach was characterized by Muskingum parameters, namely *K*, *x*, *m*, and *β*. These findings are presented in Table 3, along with performance metrics including *SSE*, *SAD*, *DPO*, and *DPOT*. The analysis of performance criteria revealed that the optimal performance was achieved when employing two sub-reaches (*NR* = 2). This was primarily attributed to the fact that NR = 2 yielded the lowest SSE values. It is noteworthy that the Muskingum parameters for *NR* = 2 were determined as follows: *K* = 0.06, *x* = 0.06, *m* = 1.46, and *β* = 0.16.

**Table 3:** Hydrologic parameters estimates and PEC values for different numbers of sub-reaches applied for Group B.

Number of sub-reaches	Model parameters				PEC			
	*x*	*K*	*m*	*β*	*SSE*	*SAD*	*DPO*	*DPOT*
1	0.12	0.08	1.60	0.39	785,220,033.00	192,284.99	4,180.37	1
2	**0.06**	**0.06**	**1.46**	**0.16**	730,213,882.59	197,004.36	3,425.99	1
3	0.02	0.05	1.37	0.10	773,926,769.66	205,472.96	2,035.31	2
4	0.00	0.04	1.29	0.07	840,716,718.70	211,969.22	1,175.80	2

Significant values are in [bold].

The calculation of performance evaluation criteria (*PEC*) values necessitates the availability of at least one year of observed flood data for the corresponding validation period. Unfortunately, for Group C, no year of observed data was available, rendering the calculation of PEC values impossible. However, the hydrologic parameters estimate provided in Table 4 can still be employed to evaluate the performance of the model. These estimates allow for comparisons between the model's predictions and those of other models. The Muskingum parameters determined using the SSA technique are presented in Table 4.

**Table 4:** Hydrologic parameters estimates and PEC values for different numbers of sub-reaches applied for Group C.

Number of sub-reaches	Model Parameters			
	*x*	*K*	*m*	*β*
1	0.19	2.49	1.33	0.05
2	0.19	1.16	1.26	0.02
3	0.16	0.76	1.21	0.01
4	0.25	0.63	1.10	0.01

For evaluation of the developed model in real field condition validation step has been considered. Tables 5 and 6 present examples of calibration simulation for Group A and B, respectively. Table 5 presents the measured inflow data from the Grand Forks USGS station, measured outflow data from the Drayton USGS station, and the corresponding routed outflow values for Group A, specifically when using a single sub-reach (*NR* = 1). The calibration years considered for Group A include 1997, 2001, 2005, 2006, 2009, 2010, and 2018. Similarly, Table 6 displays the measured inflow data from the Grand Forks USGS station, measured outflow data from the Drayton USGS station, and the associated routed outflow values for Group B. For Group B, the calibration years selected are 1999, 2004, and 2013, and these results correspond to the scenario where two sub-reaches are utilized (*NR* = 2).

**Table 5:** Calibration and calculations for single-reach Muskingum flood routing applied to Data of Group A.

Date	Q (m^3^/s)		
	Grand Forks	Drayton	Drayton *NR* = 1
3/26/2006	131.11	159.99	131.11
3/27/2006	131.39	161.41	138.33
3/28/2006	133.09	162.82	143.92
3/29/2006	139.04	164.24	148.58
3/30/2006	189.72	168.49	152.54
3/31/2006	331.31	186.89	167.42
4/1/2006	622.97	277.51	214.98
4/2/2006	1163.82	396.44	327.72
4/3/2006	1667.86	679.60	570.42
4/4/2006	1917.05	931.62	908.92
4/5/2006	2033.15	1090.20	1244.44
4/6/2006	2035.98	1319.57	1538.94
4/7/2006	1970.85	1656.54	1768.14
4/8/2006	1880.24	1979.35	1926.40
4/9/2006	1786.79	2169.07	2022.18
4/10/2006	1699.01	2217.21	2068.85
4/11/2006	1611.23	2191.72	2079.58
4/12/2006	1506.46	2135.09	2063.19
4/13/2006	1404.52	2067.13	2020.48
4/14/2006	1296.91	1976.52	1958.62
4/15/2006	1155.33	1885.90	1881.24
4/16/2006	1030.73	1783.96	1780.70
4/17/2006	928.79	1673.53	1667.06
4/18/2006	829.68	1432.83	1550.41
4/19/2006	747.56	1234.61	1431.86
4/20/2006	665.45	1127.01	1317.20
4/21/2006	588.99	1064.71	1205.38
4/22/2006	523.86	1016.57	1097.19
4/23/2006	470.06	974.10	995.03
4/24/2006	433.25	934.46	900.59
4/25/2006	396.44	883.49	817.45
4/26/2006	356.79	835.35	743.09
4/27/2006	314.32	781.54	674.65
4/28/2006	276.37	722.08	609.75
4/29/2006	250.60	662.61	548.62
4/30/2006	238.43	594.65	493.86
5/1/2006	240.41	526.69	447.85
5/2/2006	257.97	461.56	412.60
5/3/2006	282.89	424.75	390.32
5/4/2006	311.49	393.60	380.22
5/5/2006	339.80	359.62	380.66
5/6/2006	351.13	325.64	389.61
5/7/2006	351.13	297.33	400.07
5/8/2006	348.30	283.17	408.20

**Table 6:** Calibration and calculations for single-reach Muskingum flood routing applied to Data of Group B.

Date	Q (m^3^/s)		
	Grand Forks	Drayton	Drayton *NR* = 2
4/15/2013	54.65	61.45	54.65
4/16/2013	55.50	61.45	45.57
4/17/2013	57.77	63.71	50.61
4/18/2013	62.01	65.98	53.57
4/19/2013	67.96	69.94	65.15
4/20/2013	84.95	73.91	90.55
4/21/2013	102.79	83.53	114.68
4/22/2013	141.30	112.70	144.54
4/23/2013	206.15	173.58	185.68
4/24/2013	342.63	300.16	247.21
4/25/2013	484.22	424.75	326.65
4/26/2013	620.14	543.68	415.03
4/27/2013	809.86	699.43	509.28
4/28/2013	1027.90	809.86	610.92
4/29/2013	1158.16	880.65	717.68
4/30/2013	1226.12	951.45	823.28
5/1/2013	1217.62	1010.91	921.98
5/2/2013	1146.83	1059.05	1009.42
5/3/2013	1067.55	1107.19	1082.76
5/4/2013	988.26	1146.83	1140.94
5/5/2013	923.13	1183.64	1184.26
5/6/2013	860.83	1200.63	1213.71
5/7/2013	795.70	1194.97	1230.26
5/8/2013	730.57	1158.16	1234.61
5/9/2013	673.94	1121.35	1227.36
5/10/2013	620.14	1090.20	1209.21
5/11/2013	555.01	1059.05	1180.60
5/12/2013	472.89	1030.73	1141.13
5/13/2013	382.28	996.75	1089.22
5/14/2013	314.32	959.94	1022.93
5/15/2013	269.86	911.80	941.60
5/16/2013	242.96	855.17	846.81
5/17/2013	231.35	792.87	742.88
5/18/2013	232.48	722.08	637.49
5/19/2013	254.85	648.46	542.29
5/20/2013	248.91	583.33	467.36

These tables provide essential data for the calibration and validation of the respective models and facilitate the comparison between the simulated and observed flow values during the specified calibration years for each group.

Table 7 displays the validated inflow data from the Grand Forks USGS station, measured outflow data from the Drayton USGS station, and the corresponding routed outflow values for Group A in the year 2020 in Drayton, specifically when utilizing a single sub-reach (*NR* = 1).

**Table 7:** Validation and calculations for single-reach Muskingum flood routing applied to Data of Group A.

Date	Q (m^3^/s)		
	Grand Forks	Drayton	Drayton *NR* = 1
3/26/2020	156.31	163.39	156.31
3/27/2020	166.50	175.28	168.96
3/28/2020	178.68	194.25	181.50
3/29/2020	199.35	230.78	195.46
3/30/2020	250.32	291.66	215.71
3/31/2020	396.44	387.94	256.34
4/1/2020	574.83	515.37	334.13
4/2/2020	792.87	651.29	443.19
4/3/2020	993.92	753.23	579.26
4/4/2020	1197.80	815.53	729.68
4/5/2020	1407.35	863.66	891.29
4/6/2020	1512.12	920.30	1060.44
4/7/2020	1548.93	1019.41	1213.40
4/8/2020	1741.49	1169.49	1343.34
4/9/2020	2007.66	1384.69	1488.63
4/10/2020	2035.98	1650.87	1659.17
4/11/2020	1951.03	1840.60	1801.78
4/12/2020	1837.76	1970.85	1900.31
4/13/2020	1710.34	2035.98	1958.06
4/14/2020	1582.91	2055.80	1979.73
4/15/2020	1452.65	2050.14	1971.51
4/16/2020	1291.25	1979.35	1937.57
4/17/2020	1152.50	1883.07	1875.29
4/18/2020	1042.06	1781.13	1793.57
4/19/2020	948.61	1682.02	1701.19
4/20/2020	863.66	1599.90	1603.26
4/21/2020	781.54	1483.80	1502.31
4/22/2020	719.25	1345.05	1399.54
4/23/2020	662.61	1217.62	1299.62
4/24/2020	614.48	1121.35	1203.51
4/25/2020	574.83	1064.71	1112.74
4/26/2020	540.85	1005.25	1028.57
4/27/2020	504.04	937.29	951.22
4/28/2020	472.89	883.49	879.11
4/29/2020	444.57	838.18	812.79
4/30/2020	424.75	790.04	752.32
5/1/2020	399.27	739.07	698.48
5/2/2020	379.45	699.43	648.96
5/3/2020	359.62	656.95	604.36
5/4/2020	339.80	614.48	563.74
5/5/2020	319.98	574.83	526.30
5/6/2020	305.82	543.68	491.75
5/7/2020	302.99	506.87	461.61
5/8/2020	317.15	478.55	438.96
5/9/2020	325.64	455.90	426.07
5/10/2020	328.48	436.08	418.78
5/11/2020	325.64	419.09	413.89
5/12/2020	325.64	402.10	409.55
5/13/2020	322.81	387.94	406.14
5/14/2020	317.15	370.95	402.52
5/15/2020	311.49	356.79	398.02
5/16/2020	308.65	336.97	393.06
5/17/2020	305.82	325.64	388.48
5/18/2020	297.33	319.98	383.79
5/19/2020	288.83	308.65	377.55

Likewise, Table 8 presents the validated inflow data from the Grand Forks USGS station, measured outflow data from the Drayton USGS station, and the associated routed outflow values for Group B in the year 2022 in Drayton. For Group B, the model configuration involved two sub-reaches (*NR* = 2).

**Table 8:** Validation and calculations for multi-reach Muskingum flood routing applied to Data of Group B.

Date	Q (m^3^/s)		
	Grand Forks	Drayton	Drayton *NR* = 2
4/14/2022	679.60	716.42	679.60
4/15/2022	719.25	744.73	745.99
4/16/2022	722.08	773.05	808.32
4/17/2022	682.44	787.21	858.74
4/18/2022	608.81	790.04	889.08
4/19/2022	532.36	781.54	893.53
4/20/2022	461.56	753.23	870.98
4/21/2022	416.26	710.75	825.62
4/22/2022	464.40	671.11	771.37
4/23/2022	713.58	713.58	743.17
4/24/2022	1115.68	858.00	786.16
4/25/2022	1608.40	965.60	918.29
4/26/2022	1820.77	1056.22	1115.66
4/27/2022	1795.29	1203.47	1332.97
4/28/2022	1693.35	1407.35	1532.31
4/29/2022	1577.25	1622.56	1694.83
4/30/2022	1548.93	1823.60	1817.00
5/1/2022	1619.72	1976.52	1906.85
5/2/2022	1707.51	2098.28	1978.41
5/3/2022	1707.51	2191.72	2041.80
5/4/2022	1614.06	2248.36	2097.12
5/5/2022	1497.96	2254.02	2137.59
5/6/2022	1390.36	2220.04	2157.01
5/7/2022	1291.25	2157.74	2153.09
5/8/2022	1200.63	2050.14	2126.48
5/9/2022	1141.17	1968.02	2079.93
5/10/2022	1152.50	1888.73	2019.44
5/11/2022	1209.13	1823.60	1955.41
5/12/2022	1243.11	1795.29	1898.65
5/13/2022	1220.46	1826.44	1853.65
5/14/2022	1152.50	1826.44	1816.68
5/15/2022	1104.36	1840.60	1780.83
5/16/2022	1064.71	1832.10	1742.07
5/17/2022	1033.56	1795.29	1699.93
5/18/2022	1005.25	1744.32	1655.35
5/19/2022	985.43	1696.18	1609.78
5/20/2022	959.94	1682.02	1564.58
5/21/2022	931.62	1614.06	1520.30
5/22/2022	897.64	1551.76	1476.53
5/23/2022	863.66	1486.63	1432.46
5/24/2022	829.68	1427.17	1387.45
5/25/2022	792.87	1364.87	1341.14
5/26/2022	761.72	1299.74	1293.42
5/27/2022	730.57	1251.60	1244.72
5/28/2022	699.43	1214.79	1195.59
5/29/2022	673.94	1175.15	1146.56
5/30/2022	651.29	1138.34	1098.57
5/31/2022	642.79	1110.02	1053.17
6/1/2022	651.29	1076.04	1013.24
6/2/2022	682.44	1053.39	983.06
6/3/2022	707.92	1036.40	966.09
6/4/2022	713.58	1022.24	961.34
6/5/2022	702.26	1008.08	963.33
6/6/2022	682.44	991.09	965.72
6/7/2022	659.78	974.10	964.18
6/8/2022	637.13	951.45	956.82
6/9/2022	614.48	928.79	943.53
6/10/2022	591.82	900.48	925.03
6/11/2022	563.51	869.33	901.97
6/12/2022	535.19	835.35	874.55
6/13/2022	504.04	795.70	842.90
6/14/2022	487.05	753.23	808.14
6/15/2022	467.23	713.58	772.58
6/16/2022	447.41	668.28	737.80
6/17/2022	438.91	622.97	704.91
6/18/2022	430.42	580.50	675.63
6/19/2022	416.26	538.02	650.32
6/20/2022	407.76	498.38	628.00
6/21/2022	399.27	461.56	608.23
6/22/2022	399.27	427.58	591.49
6/23/2022	407.76	399.27	579.40
6/24/2022	402.10	387.94	572.20
6/25/2022	390.77	402.10	566.75
6/26/2022	421.92	413.43	563.48

Furthermore, during the evaluation of the model's performance, it was observed that the model accurately predicted the maximum outflow discharge for both Group A in 2020 (*NR* = 1) and Group B in 2022 (*NR* = 2). Specifically, for Group A, the model's prediction of the maximum outflow discharge on April 14th exhibited only a 3.7% difference compared to the measured value. Similarly, for the 2022 flood classified as Group B with *NR* = 2, the difference was 2.84%. These minimal differences indicate a close agreement between the model's predictions and the actual measured values, suggesting a high level of accuracy in the model's ability to forecast future floods.

**Figure 4:**
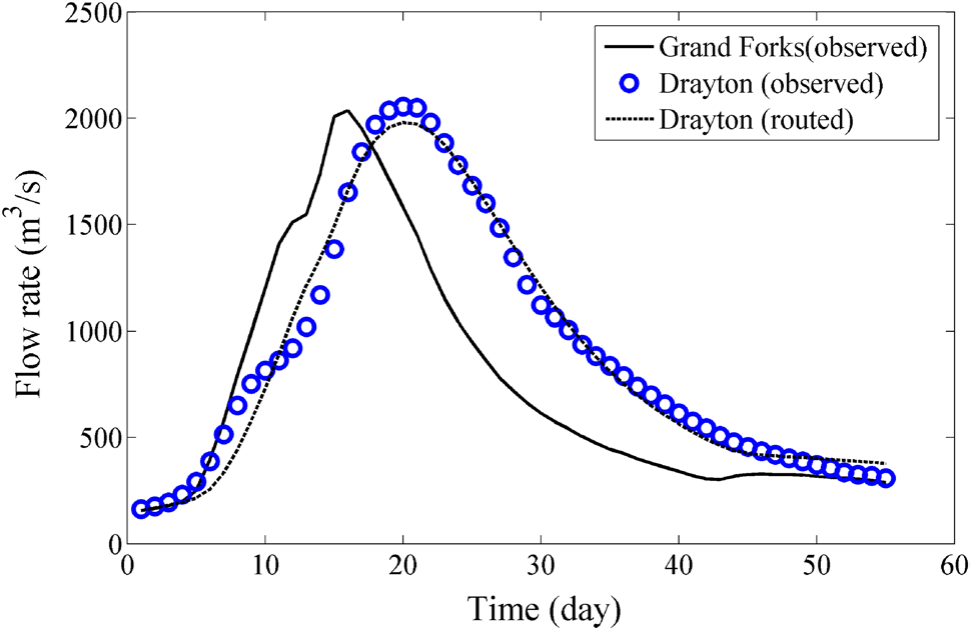
Validation for Data of Group A, 2020 flood data.

**Figure 5:**
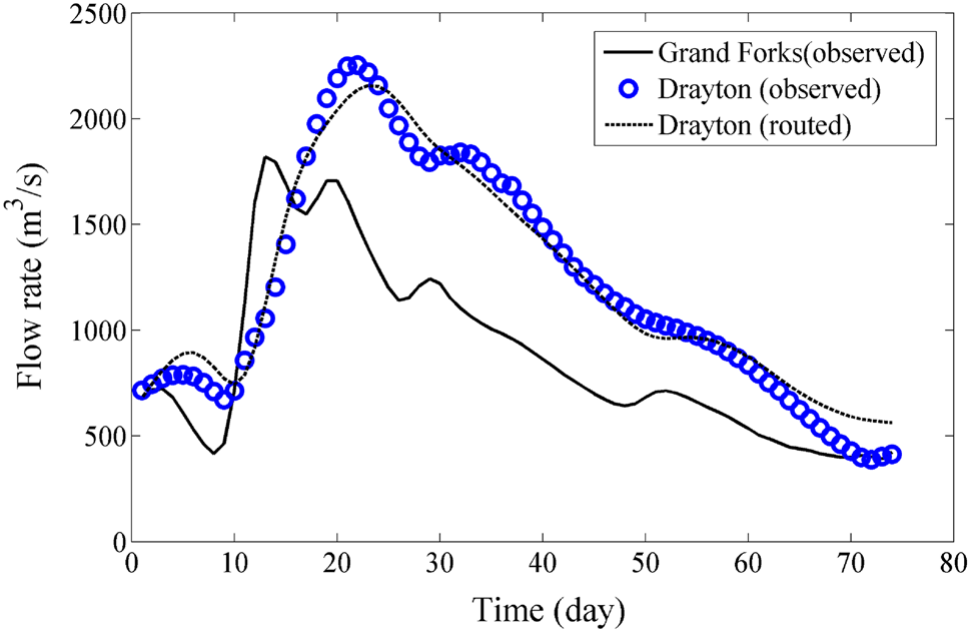
Validation for Data of Group B, 2022 flood data.

Figures 4 and 5 complement the information found in Tables 7 and 8 by presenting a visual depiction of the validation results for the flood data of Group A and B. The figures provide a graphical representation that enhances the understanding and analysis of the validation outcomes for the respective datasets.

Figures 4 and 5 serve as visual representations of the validation results for the flood data of Group A and B, respectively. These figures complement the information presented in Tables 7 and 8 by providing a graphical depiction of the validation outcomes. By utilizing visual representations, it becomes easier to comprehend and analyze the results of the validation process for each dataset. These figures display important information such as observed and simulated flood hydrographs, peak flow values, and timing of peak flows. They also show how well the simulated hydrographs match the observed data, indicating the accuracy of the routing model.

Upon careful examination of Tables 2, 3 and 4 it is evident that during the validation phase, the maximum deviation in peak time between the routed and actual outflows was only 2 units. This observation aligns with our previous experiences and findings, where we have encountered similar discrepancies when employing a dynamic wave model that considers all the necessary inputs for flood modeling^1, 34^. It is worth noting that when soft computing models are brought into the equation, such errors tend to be more pronounced.

It is essential to note that the absence of attenuation in the flood peak on the downstream side could be due to various factors. These include the absence of significant floodplain storage, a sufficiently large channel capacity, the lack of hydraulic controls such as dams or levees, and specific hydrological conditions such as continuous heavy rainfall or rapid snowmelt.

## Discussion

In our recent paper, we conducted an existing case study to assess the performance of the proposed model using the inflow-outflow hydrograph data from Wilson^60^, which represents a smooth single-peak hydrograph. The distributed Muskingum method, implemented with the *WOA* algorithm, was employed for the routing process. Table 9 provides an overview of the Muskingum model routing parameters and performance evaluation criteria (*PEC*) obtained for the nonlinear Muskingum model parameters, as described in the material and methods section of the paper. The optimal parameter values for the Wilson flood data were determined using our research technique, yielding *K* = 0.865, *x* = 0.043, *m* = 1.478, and *β* = − 0.008 for *NR* = 3.

**Table 9:** Comparison of the outflow hydrographs calculated for the Wilson flood data.

Time	Q (m^3^/s)		
	Q_in_	Q_obs_	Q_model_, NR = 3
0	22	22	22
6	23	21	21.88
12	35	21	22.52
18	71	26	25.82
24	103	34	33.13
30	111	44	43.66
36	109	55	55.38
42	100	66	66.40
48	86	75	75.44
54	71	82	81.66
60	59	85	84.53
66	47	84	83.90
72	39	80	79.91
78	32	73	73.05
84	28	64	64.10
90	24	54	54.16
96	22	44	44.37
102	21	36	35.82
108	20	30	29.24
114	19	25	24.74
120	19	22	21.89
126	18	19	20.15

Furthermore, we analyzed the impact of varying the number of sub-reaches (*NR*) on the model's performance. The maximum outflow discharge, occurring at the 60th hour of the flood data, was examined for *NR* values ranging from 1 to 5. The results showed that the difference in peak discharge varied as follows: − 1.67%, 1.01%, 0.13%, 1.18%, and 0.96% for *NR* = 1 to *NR* = 5, respectively. Notably, *NR* = 3 exhibited the lowest difference in peak discharge compared to the Wilson flood data.

It is clear from comparing the findings of this case study with prior studies that the proposed model demonstrates consistent accuracy in predicting peak discharge across different datasets. Both the Group A and Group B case studies, along with the Wilson flood data, indicate close agreement between the model's predictions and the observed values. This reaffirms the model's reliability and its potential for effectively predicting future floods.

In comparison to the previous studies, our research offers distinctive findings. While Ayvaz and Gurarslan^61^ introduced a novel partitioning approach for flood routing models, our study focuses on analyzing the impact of varying the number of sub-reaches on the performance of the nonlinear Muskingum model. In contrast to the primary emphasis of Hirpurkar and Ghare^62^ on parameter estimation, our research evaluates the accuracy of peak discharge predictions using the proposed model. Furthermore, while Barbetta et al.^63^ addresses river discharge estimation and rating curve development, our study concentrates on peak discharge prediction and the model's reliability across diverse datasets. Importantly, our study distinguishes itself by utilizing a specific case study with real flood data, providing a unique and valuable contribution to the field. By analyzing actual data, our findings offer practical insights, showcasing the effectiveness of the proposed model in real-world scenarios. This emphasis on practicality enhances the applicability and relevance of our research.

## Conclusion

This study aims to develop and evaluate a nonlinear Muskingum model for river modeling, with a specific focus on enhancing accuracy through the incorporation of lateral inflow. The Grand Forks and Drayton USGS stations serve as case studies for parameter estimation using the distributed Muskingum method. The results demonstrate that the developed nonlinear Muskingum model effectively routes floods through these stations. Our selection of this area is rooted in a prior spatial analysis we conducted, which highlighted a particular region's vulnerability. This area, situated between Grafton city, Grand Forks, and Emerson, demonstrated a high susceptibility to severe floods when we examined the permanent water area (PWA) and seasonal water area (SWA). Building upon these previous findings, our current research hones in on this specific locale, aiming to delve deeper into flood dynamics and comprehensively grasp the underlying factors that make it exceptionally vulnerable^42^.

Through an analysis of hydrologic parameters and model performance, the study underscores the significant impact of the number of sub-reaches on parameter estimation and modeling precision. Optimal model performance varies between case studies, underscoring the importance of selecting the appropriate number of sub-reaches for precise peak discharge predictions.

For Group A, where snowmelt is the primary driver with minimal rain-on-snow impact, the findings indicate that a single sub-reach provides the best performance in accurately predicting peak discharge. This suggests that a simplified representation of the river system adequately captures the routing characteristics of this flood type. In contrast, for Group B, representing flood events primarily characterized by rain-on-snow conditions, the study reveals that employing two sub-reaches optimizes model performance. This implies that considering additional sub-reaches is essential for accurately capturing the routing dynamics and behavior of this specific flood type.

The findings hold practical significance for flood prediction and management, offering valuable insights to decision-makers and stakeholders involved in flood mitigation. The proposed model holds potential for broader application beyond the specific case studies, contributing to enhanced river system modeling and flood management practices. For future studies, non-linear Muskingum model and Muskingum-Cunge approach should be compared in real field case studies to better understand their abilities compare to each other. Moreover, β parameter of the proposed model can be consider variable to examine effects of its variation on the model prediction performance.

## Data Availability

Some or all data, models, or code that support the findings of this study are available from the corresponding author upon reasonable request. These data include flood data for Drayton and Grand Forks.
